# Investigating the Viability of Fat Cells Over 1, 3, and 6 Months After Freezing at −18°C

**DOI:** 10.1111/jocd.70160

**Published:** 2025-04-15

**Authors:** Mehdi Rasti, Hamidreza Piri Ardekani, Hossein Mirhendi, Mohadeseh Mofidi, Leila Dehghani, Vajihe Azimian Zavareh

**Affiliations:** ^1^ Department of Plastic Surgery Isfahan University of Medical Sciences, Medical Education Research Center Isfahan Iran; ^2^ Core Research Facilities Isfahan University of Medical Sciences Isfahan Iran; ^3^ Department of Medical Parasitology and Mycology School of Medicine, Isfahan University of Medical Sciences Isfahan Iran; ^4^ Department of Pathology School of Medicine, Isfahan University of Medical Sciences Isfahan Iran; ^5^ Department of Tissue Engineering and Applied Cell Sciences School of Advanced Technologies in Medicine, Shahid Beheshti University of Medical Sciences Tehran Iran; ^6^ Department of Plant and Animal Biology Faculty of Biological Sciences and Technology, University of Isfahan Isfahan Iran

**Keywords:** adipocyte cells, fat tissue, fresh, frozen, liposuction, viability

## Abstract

**Background:**

Lipofilling is a natural, low‐risk, and long‐lasting method for filling, reconstructing, and improving soft tissues such as the face, with minimal discomfort for patients. Many plastic surgeons prefer autologous fat grafting in aesthetic surgery due to its availability, cost‐effectiveness, biocompatibility, and absence of allergic and carcinogenic concerns. Despite the advantages of autologous fat injection, one of the main drawbacks is the variable persistence of injected fat tissue. Given the significant implications of this issue in advanced countries, this study aims to investigate the survival of fat cells after freezing at different time intervals (1, 3, and 6 months).

**Methods:**

Thirty female participants were enlisted for this research, and the viability of fat cell specimens was assessed at intervals of 0, 1, 3, and 6 months post‐freezing at −18°C. The evaluation of viable adipocytes was conducted using the XTT assay, a live/dead staining method using fluorescence microscopy after staining with fluorescein diacetate (FDA) and propidium iodide (PI), along with histological analysis of fat tissue after freezing at the indicated time intervals.

**Results:**

The results showed that the viability of frozen fat samples decreases by 34%, 60%, and 80% after 1, 3, and 6 months, respectively, compared to non‐frozen samples on Day 0.

**Conclusions:**

The findings of this study underscore a rapid decline in adipocyte viability after storage at −18°C at different time intervals (1, 3, and 6 months), at which points only around 60%, 40%, and 20% of fat cells remained viable, respectively. These results suggest that current fat preservation techniques utilizing either a −18°C freezer are not sufficient for maintaining the long‐term viability of adipocytes, and alternative cryopreservation methods are needed to preserve fat cells.

## 1 Introduction

The autologous fat grafting technique is a successful and convenient approach used to enhance soft tissues in cosmetic and reconstructive plastic surgery, as well as in cosmetic treatments and in improving scar tissue at the surgical site for breast cancer patients following radiotherapy [1]. It is distinguished by the absence of foreign body or immune reactions and presents multiple donor site options, making it a favored choice in plastic and reconstructive surgery. Consequently, its clinical applications are constantly increasing [2, 3]. However, the key drawback of the procedure is the challenge in accurately predicting the survival rates of the transplanted fat. This uncertainty arises from the potential for absorption or necrosis to influence varying quantities of adipose tissue after autologous fat transfer [4].

The survival of grafted fat cells varies widely, ranging from 30% to 80%, depending on various factors. These factors include the donor site for fat cell harvesting, the storage method of fat cells (freezing or not), the technique and location of cell injection, grafting interval, and methods of analysis [5]. The diverse nature of these differences has presented challenges in comparing studies. Different techniques, including overcorrection and retransplantation, have been employed to tackle the high absorption rates of adipose tissue. However, overcorrection may result in unrealistic cosmetic effects, while retransplantation involves further surgical procedures. Recent studies have explored the possibility of freezing or cryopreserving excess adipose tissue post‐transfer for potential reuse in cases where retransplantation is necessary [6]. The survival rate of adipocytes when using frozen or cryopreserved fat is crucial, as it significantly influences the successful engraftment of adipose tissue following reinjection [7]. Studies have indicated that cells maintain a degree of metabolic activity when they are partially frozen at −20°C, which is the typical storage temperature for commercial freezers [8, 9]. Some scientists suggest that adipose‐derived fat cells from regular liposuction can be preserved at low temperatures, but freezing fat may lead to a significant reduction in viable fat cells, as indicated in certain studies [10, 11]. Currently, the typical storage temperature for fat is around −20°C and previous studies indicate variable survival times for fat cells stored at this temperature [12, 13]. Previous studies investigating the viability of cryopreserved fat tissues over time have produced varying results. Schuller‐Petrovic found that slowly freezing the tissue to −20°C immediately after harvesting did not harm the adipocytes [14]. Sommer and Sattler discovered live adipocytes even after cryopreserving at −20°C for 3 years [15]. Conversely, Wolter et al. observed adipocyte destruction after 48 h of freezing at −20°C, leading to mostly non‐viable cells upon reuse [16]. These conflicting findings have caused uncertainty regarding the impact of cryopreservation on adipose tissues.

Lipofilling, which refers to filling with fat, is a natural, low‐risk, and long‐lasting method for filling and reconstructing soft tissues such as the face with minimal discomfort for patients. Today, many plastic surgeons prefer autologous fat grafting for soft tissue filling in cosmetic surgery due to its availability, cost‐effectiveness, non‐allergenic and non‐carcinogenic properties, and biocompatibility [17]. Considering that our surgical team's observations of autologous fat injection even 6 months after freezing have shown acceptable evidence of tissue regeneration, we decided to examine the viability of frozen fat cells at 1, 3, and 6 months post‐freezing at −18°C in a common freezer. This study aims to determine whether the observed sustainability of clinical effects is due to the presence of live fat cells or other factors.

## 2 Material and Methods

### 2.1 Fat Processing and Freezing

The study sourced samples from individuals undergoing tumescent liposuction or lipoaugmentation (fat transfer) procedures. Approval for the study was secured from Hospital. Patients were informed about the study objectives and voluntarily contributed surplus fat, considered waste material, from their tumescent procedures. To ensure ethical practices, enrolled participants signed an informed consent form that contained a clause regarding sample donation. All specimens and associated information received by Hospital for Medical Research were handled anonymously to protect patient privacy. All procedures were done under the ethical committee.

Sampling was done from all patients referred to the surgery clinic of Al‐Zahra Hospital (S) who met the inclusion criteria. The fat graft was harvested under general anesthesia. Prior to the harvesting procedure, a solution of 0.2% lidocaine with epinephrine (1:400000) was injected to minimize pain and reduce bleeding. Fat was then harvested using a two‐hole Coleman harvesting cannula attached to a 10‐cc syringe. Sample collection was done from mid‐2022 to early 2023. During this period, 13 samples were collected and studied. The inclusion criteria were women with an age range: 18–69 years, and willingness to participate. Individuals with diabetes, HIV/AIDS, immune system deficiencies, and those taking immunosuppressive medications were excluded. Each patient's sample is divided into four parts—one for control (Day 0) and three for freezing in different time periods: 1, 3, and 6 months.

Of the 13 samples acquired through tumescent liposuction, 8 were obtained from the hips and thighs, and the remaining samples were taken from the waist, abdomen, neck, and upper arms. First, 15 mL of fat was taken from the patient through liposuction and collected in a sterile tube, and transferred to the laboratory. The fat was washed with large volumes of phosphate buffer saline (PBS^−^, Bio Idea, Iran) + penicillin/streptomycin (Bio Idea, Iran), followed by centrifugation at 100 × *g* for 3 min. The cleaned fat was washed again with PBS^−^, and the floating, clean fat was recovered by centrifugation. Next, the washed fat was aliquoted into four Falcon 15 mL tubes, three Falcons were stored in a −18°C freezer, and one Falcon was used for the enzymatic digestion process. For this reason, the fat tissues were digested with 3 mL of 0.5 mg/mL collagenase Type I solution (Solarbio, #C8140, China) in DMEM medium at 37°C for 40 min with frequent swirling and mixing to release the adipocytes. Then, 15 mL complete DMEM (Bio Idea, Iran) containing 10% FBS (Bio Idea, Iran) was added to terminate the digestion. The treated samples were filtered using a 100‐μm strainer (PluriSelect, #43–50 100‐51, Germany) and fat cells were collected after centrifugation at 1000 rpm for 5 min.

The frozen samples were removed from the freezer after 1, 3, and 6 months and allowed to thaw at laboratory temperature for 20 min. After thawing, they underwent an enzymatic digestion process similar to the one described above. For transplantation, the fat is transferred to a 1‐cc syringe for placement in the face and hands, or a 3‐cc syringe for placement in the breasts or body. The syringe is then connected to a blunt cannula and fat is injected under the skin in the targeted area.

### 2.2 Analysis of the Isolated Fat Cells

#### 2.2.1 XTT Assay

The metabolic activity was assessed using the XTT assay in all samples immediately after harvesting and again at 1, 3, and 6 months of storage at −18°C. The cell Proliferation Assay XTT powder (Sigma, X4626, USA) was used and dissolved in culture media (4 mg in 4 mL media) to measure cell viability. For preparing the XTT detecting solution, 10 μL of the PMS (Phenazine methosulfate, 3 mg in 1 mL PBS^−^) solution (Sigma, #P9625, USA) was added to the 4 mL of XTT solution to create the detection solution. One hundred microliter of isolated fat cells was transferred to each well of 96‐well plates (3 wells for each sample). Then 50 μL XTT detecting solution was added to each sample in 96‐well plate and incubated at 37°C for 3 h, and the absorbance was measured at 450 nm using an ELISA reader (Stat Fax 2100, USA). All values were presented as relative measurements in comparison to the fresh samples. The results from the fresh samples were expressed as a percentage, and the metabolic activity of the viable cells was evaluated.

#### 2.2.2 Viability Staining of Fat With Fluorescein Diacetate (FDA) and Propidium Iodide (PI)

After the specimens were treated with collagenase and filtered using a 100‐μm strainer, 150 μL of isolated adipocyte cells were added to a vial 1.5 mL. Twelve micromolar of FDA (Solarbio, #F8040, China) and 3750 nanomolar of PI (Sigma, #P4170, USA) were prepared in PBS and added to the adipocytes in equal volume, and mixed by inverting three times. After 5 min, 30 μL of the adipocytes were placed on a clean microscope slide and coverslips were added. Specimens were examined under ultraviolet illumination in a fluorescence microscope (Olympus BX61, Japan) using a triple‐pass emission. Viable adipocytes emitted a bright green fluorescence, especially along their periphery, while the dead cells appeared black. The nuclei of the deceased cells showcased a red fluorescence due to the absorption of propidium iodide. The analysis involved assessing three different fields of view, and the average number of stained cells was compared before and after the freezing process.

#### 2.2.3 Histological Analysis

The thawed adipose tissue (1 mL) was fixed in a 10% paraformaldehyde solution for 20 min and subsequently placed in a 15% sucrose solution using a horizontal shaker for 2–3 h. The samples underwent dehydration with 30% sucrose for 12 h and were then rapidly frozen after being embedded in optimal cutting temperature solution. A microtome was employed to produce cryopreserved sections, using all odd‐numbered slides for histological analysis and staining with hematoxylin and eosin. After staining with hematoxylin and eosin, mature fat cells with intact nuclei were counted under a microscope. Additionally, samples stored in a freezer at −18°C were re‐stained and re‐evaluated for cell count after 1, 3, and 6 months. The freezer maintained a constant temperature of −18°C, essential for the preservation of the adipose tissues.

### 2.3 Statistical Analysis

All data in this study were presented as mean ± standard deviation. Statistical analysis was conducted using the Mann–Whitney *U‐*test, with a *p* value of ≤ 0.05 deemed statistically significant.

## 3 Results

### 3.1 XTT Assay

**FIGURE 1:**
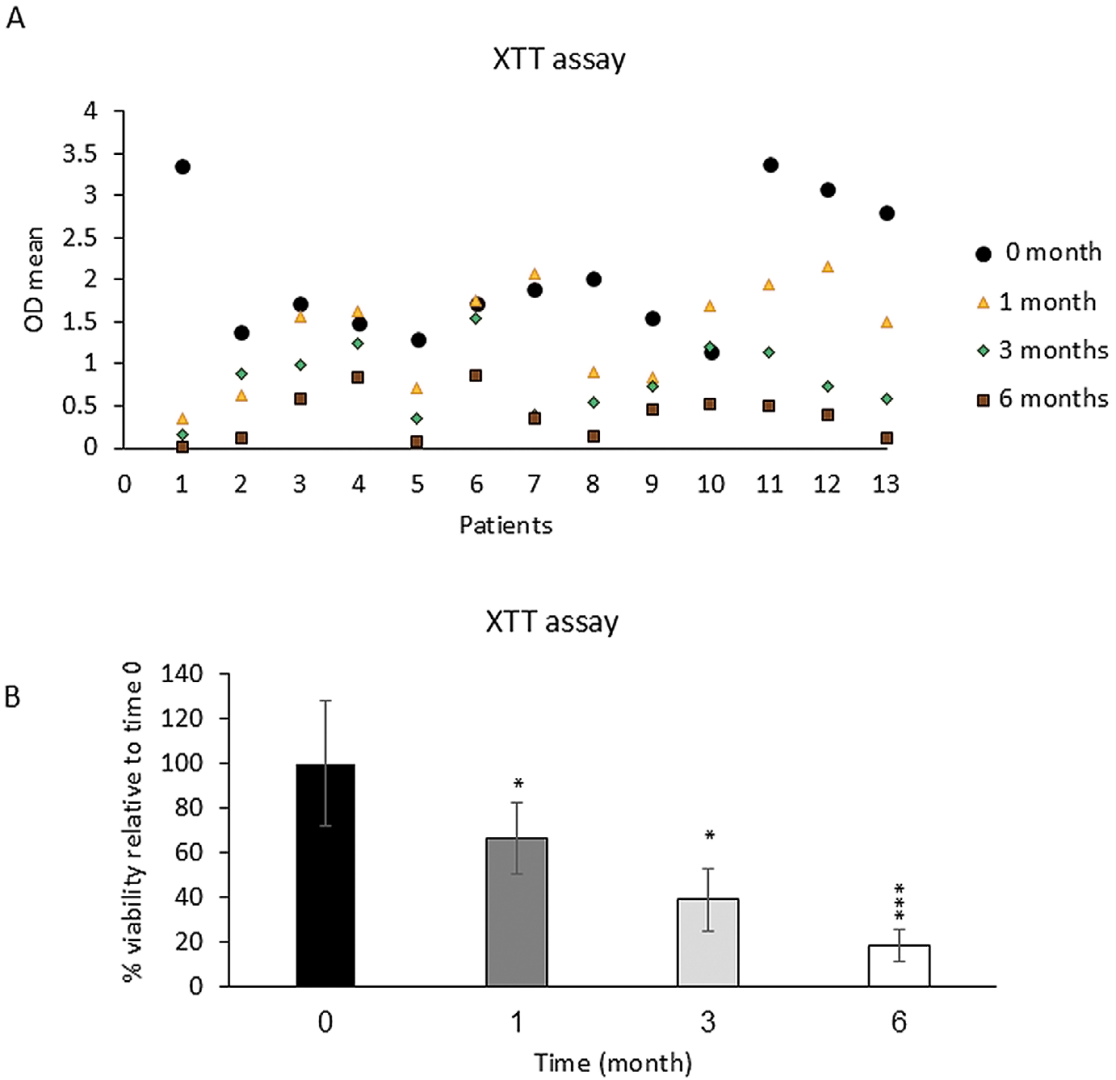
Changes of enzymatic activity after freezing to −18°C and storage for 1, 3, and 6 months. XTT assay in all adipocyte samples in fresh and in 1, 3, and 6 months' frozen samples was done. (A) Metabolic activity of all samples as fresh and 1, 3, and 6 months frozen was assessed by XTT assay and shown as OD mean. (B) Mean viability percentage of frozen samples in comparison to fresh samples was shown. Data are presented as the mean ± SD performed in triplicate. *p < 0.05, **p < 0.01, ***p < 0.001.

Adipocytes' metabolic activity, as assessed by the XTT assay, indicated that fresh samples exhibited 100% activity. In these samples, the mean OD before freezing was 2.06 ± 0.28 and after freezing for 1, 3, and 6 months was 1.36 ± 0.16, 0.8 ± 0.14, and 0.38 ± 0.07, respectively (Figure 1A). There was a significant decrease in the viability of frozen samples stored at −18°C, with reductions of 34% (*p* < 0.05), 59.2% (*p* < 0.05), and 81.31% (*p* < 0.001) after 1, 3, and 6 months, respectively, indicating a statistically significant decrease in metabolic activity compared to fresh samples (Figure 1B).

**FIGURE 2:**
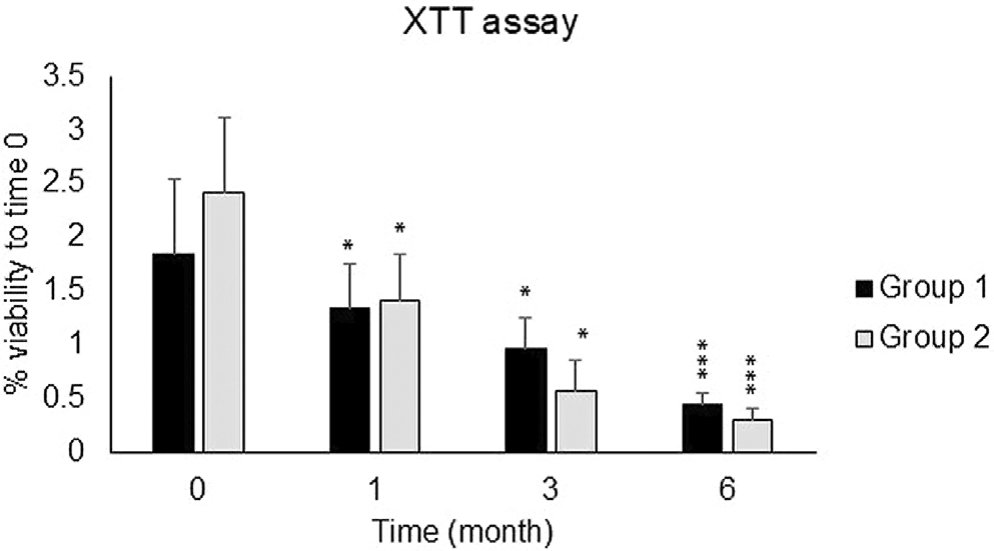
Metabolic activity of frozen samples during 1, 3, and 6 months after freezing compared to fresh samples between 2 groups based on the site of sampling (Group 1: hips and thighs and Group 2: other regions). Data are presented as the mean ± SD performed in triplicate. *p < 0.05, **p < 0.01, ***p < 0.001.

**FIGURE 3:**
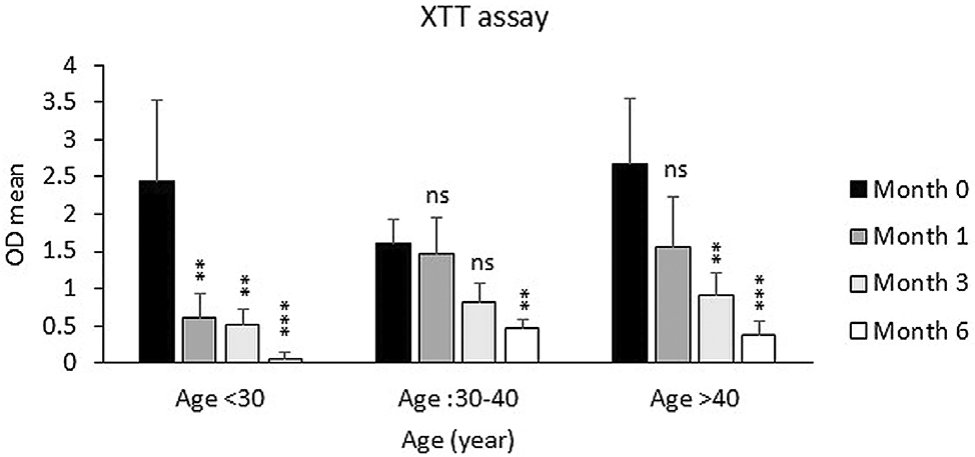
Metabolic activity of frozen samples during 1, 3, and 6 months after freezing compared to fresh samples between different age groups (age < 30, age 30–40, and age > 40). Data are presented as the mean ± SD performed in triplicate. ns: Not significant. *p < 0.05, **p < 0.01, ***p < 0.001.

The samples were also categorized into two groups based on the sampling location (Group 1: hips and thighs and Group 2: other sites), and the viability of fat cells was examined depending on the sampling site. As shown in Figure 2, the viability of Group 2 was lower than that of Group 1 after 3 and 6 months, but this decrease was not significant. Also, the decrease in the percentage of frozen fat cell viability in the age groups under 30 and over 40 was much higher than in the 30–40 age group specifically after 1 and 3 months of freezing in comparison to fresh samples (Figure 3).

### 3.2 Fat Cell's Viability Assessment by FDA Staining

**FIGURE 4:**
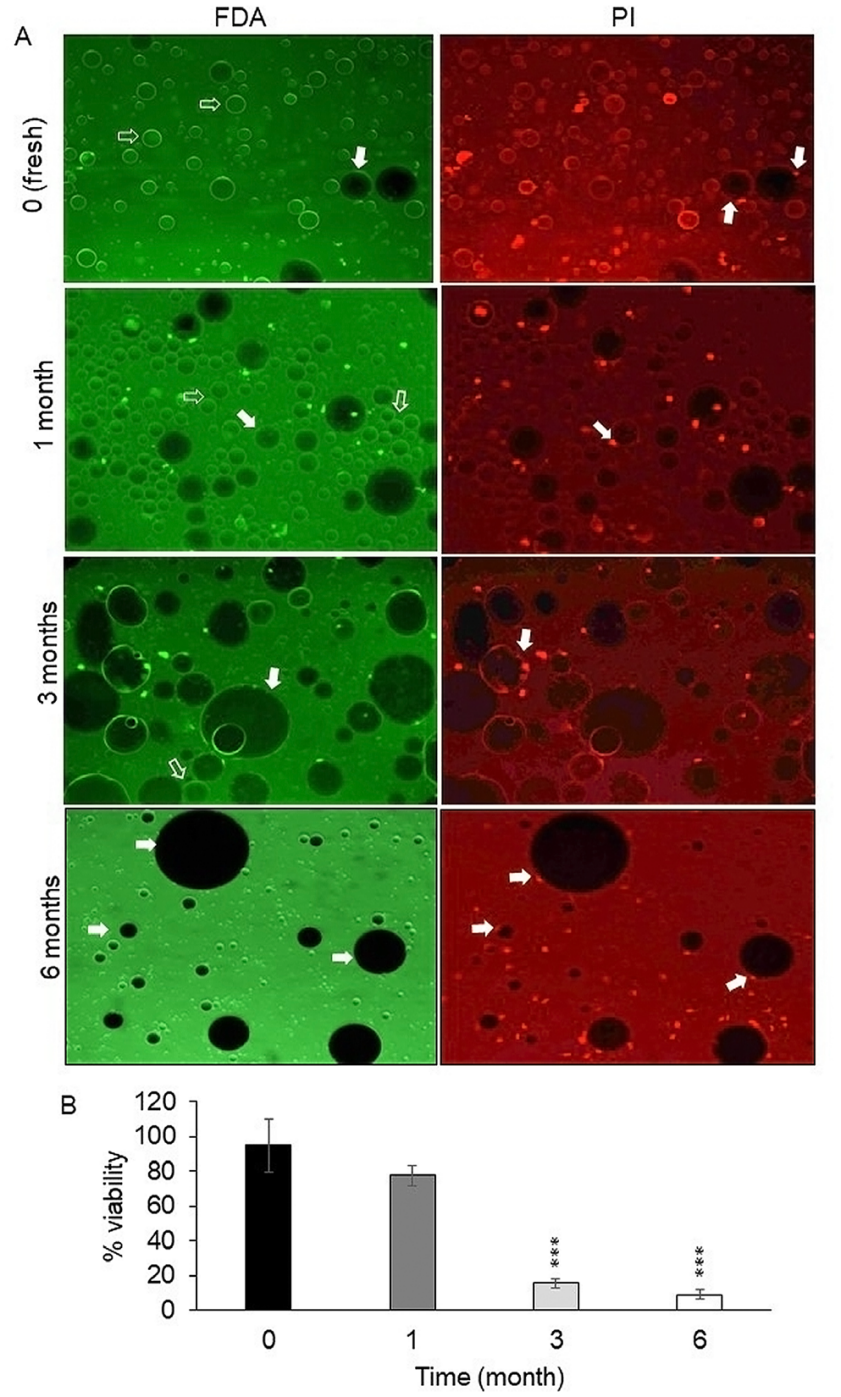
Representative view of adipocytes in single cell suspension and FAD/PI staining. (A) Fresh specimen, after freezing to −18°C for 1, 3, and 6 months later. Dark cells indicate damaged cells (Scale bar: 100 μm). (B) Quantitative evaluation of the percentage of stained‐viable cells in fresh and frozen samples after 1, 3, and 6 months freezing in −18°C. *p < 0.05, **p < 0.01, ***p < 0.001.

After the assessment of cell viability by XTT assay, staining of fat cells with FDA/PI was done. A representative view of the fluorescence‐stained adipocytes in fresh and frozen is shown in Figure 4A. Empty white arrows denote viable adipocytes, while solid white arrows point to nuclei stained with propidium iodide, indicating damaged and dead cells. The viable cells that were fresh and those stored at −18°C for 1 month exhibited a brighter green fluorescence compared to the viable cells that had been frozen at −18°C for 3 and 6 months (Figure 4A). Our data showed that the adipocyte viability of the fresh fat tissues was 95.7%. After freezing at −18°C for 1, 3, and 6 months, we found a reduction in adipocyte viability to 76.3%, 17.5%, and 14.6%, respectively. A statistically significant reduction was observed when comparing the fresh samples to those that had been frozen for 3 and 6 months (*p* < 0.001) (Figure 4B).

### 3.3 Histological and Morphological Examination of Fresh and Frozen Adipose Tissue

**FIGURE 5:**
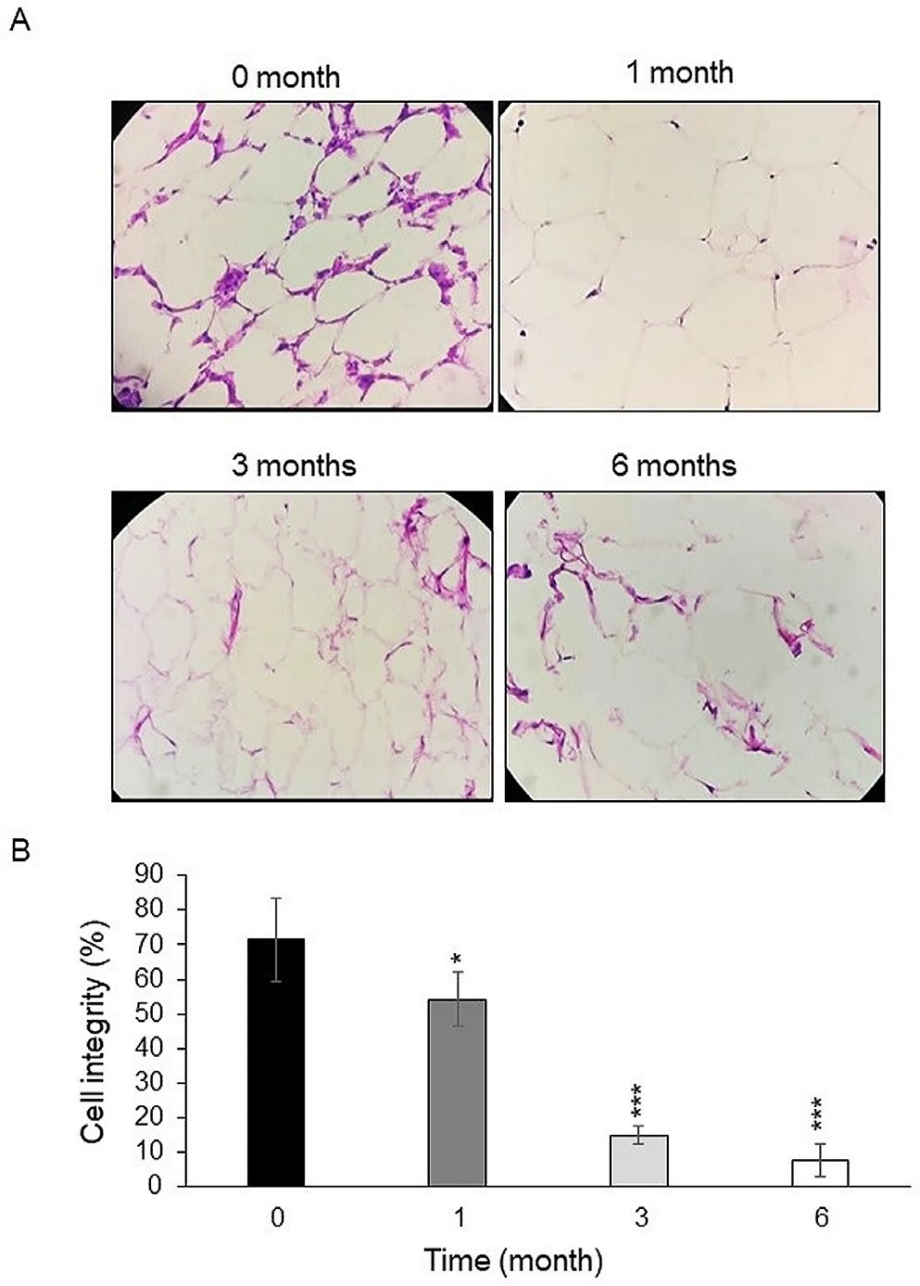
H&E staining of the fresh and frozen fat tissue (original magnification 200×). (A) Tissue morphology of adipocytes in fresh and frozen samples after 1, 3, and 6 months. (B) Quantitative analysis of adipocyte cell integrity in frozen groups relative to fresh samples. H&E, hematoxylin and eosin. *p < 0.05, **p < 0.01, ***p < 0.001.

Under a 200 × microscopic field, we observed that the adipocytes were organized in a honeycomb‐like pattern (Figure 5A), characterized by their small volume, distinct and intact cell membranes as well as intercellular substances, and a uniform shape. In the frozen groups (Figure 5A), the margins of the adipocyte membranes were less defined and showed a reduced rounding, with some membranes appearing distorted or damaged. Cell integrity was worse in frozen groups (1, 3 and 6 months after freezing) than in the fresh group (54.12% (*p* < 0.05), 15% (*p* < 0.001), and 7.8% (*p* < 0.001) vs. 71.42% respectively, Figure 5B).

## 4 Discussion

The study analyzed the viability of fresh and frozen adipocytes at a temperature of −18°C using the XTT assay, fluorescence staining (FDA/PI) and histological analysis. Frozen fat exhibited a rapid decline in adipocyte viability to below 66%, 40.8%, and 19% on the 1, 3, and 6 months of freezing, irrespective of the temperature, suggesting that −18°C can be used for adipocyte cell preservation. The unique characteristics of mature human adipocytes, including their limited proliferation and adherence to culture conditions, pose challenges in accurately assessing their viability compared to other cell types. This study highlights the complexity of preserving and evaluating the viability of mature adipocytes, emphasizing the need for specialized approaches in this field.

Various techniques are commonly employed to study the survival rates of adipocytes [18], including live cell counting with trypan blue staining, measurement of enzyme activity using glycerol‐3‐phosphate dehydrogenase analysis, assessment of mitochondrial activity through XTT or MTT assays, and observation of cell morphology using hematoxylin and eosin staining [13, 19]. Previous studies have also utilized volume measurement of the fat layer, counting live adipocytes with trypan blue staining, and evaluating mitochondrial activity. However, our study not only employed these established techniques but also integrated them into a comprehensive framework that provides a more robust assessment of adipocyte viability over time. In a study conducted by Eto et al. trypan blue staining was utilized to assess the survival rate, followed by stem cell isolation and culture of adipose‐derived stem cells (ADSC) from frozen fat tissue [13, 19, 20, 21, 22]. The study notably found that allowing natural thawing at room temperature resulted in a greater preservation of viable cells, reducing adipocyte damage [10, 16]. Schuller‐Petrovic [14] indicated that slowly freezing the tissue to −20°C soon after harvesting did not adversely affect the adipocytes. Likewise, Sommer and Sattler [15] reported the presence of live adipocytes even after cryopreservation at −20°C for 3 years. Nonetheless, Wolter et al. [16] reported that adipocytes were damaged after 48 h of freezing at −20°C, indicating that the reuse of adipose tissues cryopreserved at this temperature primarily yields injections of dead cells. These conflicting findings have caused confusion regarding the impact of cryopreservation on adipose tissues. The current study demonstrated a significantly higher average number of viable fat cells in fresh samples compared to those frozen for less than 7 months, in contrast to samples frozen for more than 8 months, with the difference being statistically significant.

Liu et al. [10] reported varying degrees of metabolic activity in fat cells stored at −20°C, indicating variable cell survival at this temperature, which was consistent with our results. On the other hand, Schuller‐Petrovic et al. found that slow freezing of fat tissue at −20°C immediately after suction has no detrimental effect on fat cell viability [14].

Studies by Sommer et al. [15] demonstrated viable fat cells in fat stored at −20°C even after 3 years. However, Wolter et al. stated that fat cells are destroyed after 48 h of storage at −20°C, and injecting fat cells stored in a freezer after 48 h results in a significant number of dead cells [16].

Unlike many studies that limit their scope to shorter time frames or single observation points, our research provides a comprehensive evaluation of adipocyte viability over 1, 3, and 6 months of freezing at −18°C. This extended analysis offers deeper insights into the long‐term effects of freezing on fat cell viability, which is crucial for improving clinical applications in fat grafting and storage.

Present research employed a multi‐faceted approach to assess adipocyte viability, using XTT assays to measure metabolic activity, fluorescence staining to distinguish live and dead cells, and histological analysis to examine structural integrity. This combination of methods delivers a more thorough and accurate evaluation of cell viability than studies that rely on just one technique, thereby enhancing the reliability of our results. Our focus on freezing at −18°C—a temperature that standard commercial freezers can easily maintain—ensures that our findings are more practical and accessible for a wider range of clinical and laboratory environments. This contrasts with studies that use lower, less accessible cryopreservation temperatures, making our research directly applicable to everyday practices, where advanced freezing equipment may not be available.

In our study, fat samples were frozen at −18°C in a standard freezer without additional cryoprotectants or advanced freezing protocols commonly used in tissue banks. It showed that although the freezing of fat tissue leads to a reduction in the percentage of viable cells over time, a portion of the cells remains alive. The repair processes and the clinical effects observed from the injected fat may be associated with this small percentage of viable cells. Nevertheless, further studies in this area are necessary. This method was selected to assess the viability of fat cells under conditions that might be encountered in routine clinical settings, where such facilities may not always be available. Accredited tissue banks, in contrast, typically use cryopreservation protocols that involve controlled‐rate freezing and cryoprotective agents to minimize ice crystal formation and cellular damage, ensuring higher post‐thaw viability of tissues.

### 4.1 Limitations

Despite the significant findings, this study has some limitations, including a small number of adipose tissue samples due to patient withdrawals, lack of adequate fat samples in some patients, and the time required for re‐sampling. Additionally, the lack of molecular tests, such as apoptosis analysis in fat cells, restricts the depth of the evaluation. Future research with a larger cohort and the incorporation of molecular methods will help strengthen the conclusions and provide a more comprehensive assessment of graft viability and outcomes.

## 5 Conclusions

In conclusion, this study underscores the complexities and challenges associated with the preservation and viability assessment of mature human adipocytes. The significant decline in cell viability observed in adipocytes frozen at −18° cover time highlights the need for careful consideration of freezing duration and method. While various techniques exist to evaluate adipocyte survival, the inconsistent findings from previous studies regarding the effects of cryopreservation at different temperatures—particularly at −20°C—suggest that further research is necessary to standardize protocols and clarify the optimal conditions for adipocyte preservation. Overall, this study emphasizes the importance of specialized strategies to enhance the viability of mature adipocytes, paving the way for more effective tissue engineering and regenerative medicine applications.

## Conflicts of Interest

The authors declare no conflicts of interest.

## Data Availability

The data that support the findings of this study are available from the corresponding author upon reasonable request.
